# 

**Published:** 1908-08-08

**Authors:** 


### CONTENTS.

PAGE

**The Care and Control of the Feeble-minded** ... 485
**The Registration of Deaths Bill** ... ... ... ... ... 486
**Annotations—**
Workmen's Compensation Acts—Livingstone College—Overlaying ... ... ... ... ... ... ... ... ... 487
**Medical Opinion and Movement** ... ... ... ... 488
**Hospital Clinics—**
Sick-room Cookery. By Professor Robert Saundby, M.D., LL.D., F.R.C.P. ... ... ... ... ... ... ... 491
**Special Article—**
The Care of the Children During a Hot Summer ... ... 493
**Medicine—**
Enlargements of the Spleen—III. ... ... ... ... ... ... 495
Myocarditis and Myocardial Diseases—I. ... ... ... ... 496
**Surgery—**
Œsophageal Obstruction.—II. ... ... ... ... ... ... ... 497
Some Aspects of Intestinal Obstruction ... ... ... ... 498

PAGE

**Life Assurance—**
Practical Hints upon Medical Examination for Life Assurance—II. ... ... ... ... ... ... ... ... ... 499
**Dermatology—**
Treatment of Ringworm of the Scalp by Means of the X-Rays 500
**The Royal Army Medical Corps Section—**
The Territorial Army and Camp Sanitation ... ... ... 501
**Therapeutic Notes—**
The Need for Uniformity in the Quality of Drugs ... ... 502
**The Practitioner's Relaxations and Hobbies—**
Golf and Physique... ... ... ... ... ... ... ... ... 503
**Mental Diseases—**
Delusions ... ... ... ... ... ... ... ... ... ... ... 504
**Hospital Administration—**
Metropolitan Hospital Sunday Fund ... ... ... ... ... 505
**Nursing Administration—**
Wanted, a Constructive Policy ... ... ... ... ... ... 508
**News and Coming Events** ... ... ... ... ... ... 509
**A Dialogue in Blank Verse** ... ... ... ... ... ... 510

